# Asymmetric Collagen/chitosan Membrane Containing Minocycline-loaded Chitosan Nanoparticles for Guided Bone Regeneration

**DOI:** 10.1038/srep31822

**Published:** 2016-08-22

**Authors:** Shiqing Ma, Aidina Adayi, Zihao Liu, Meng Li, Mingyao Wu, Linghao Xiao, Yingchun Sun, Qing Cai, Xiaoping Yang, Xu Zhang, Ping Gao

**Affiliations:** 1 School and Hospital of Stomatology, Tianjin Medical University, 12 Observatory Road, Tianjin 300070, PR China; 2 The Key Laboratory of Beijing City on Preparation and Processing of Novel Polymer, Beijing University of Chemical Technology, Beijing 100029, PR China

## Abstract

Infections caused by pathogens colonization at wound sites in the process of bone healing are considered as one of the major reasons for the failure of guided bone regeneration (GBR). The objective of this study was to prepare a novel asymmetric collagen/chitosan GBR membrane containing minocycline-loaded chitosan nanoparticles. The morphologies of the membranes and nanoparticles were observed by SEM and TEM, respectively. The characterization and biocompatibility of the membranes was evaluated. The effect of the membrane on bone regeneration was assessed using the critical-size at cranial defect model. TEM images showed the spherical morphology of the nanoparticles. The results of SEM indicated that the asymmetric membrane contained a dense collagen layer and a loose chitosan layer. An *in vitro* experiment showed that the membrane can inhibit bacterial growth and promote osteoblasts and fibroblasts growth. The membrane showed the ability to promote angiogenesis and enhance bone regeneration *in vivo*. An asymmetric collagen/chitosan GBR membrane can be fabricated by loading minocycline encapsulated chitosan nanoparticles, and shows satisfactory biocompatibility and barrier function, which enhances bone regeneration. Therefore, this antibacterial GBR membrane is a promising therapeutic approach to prevent infection and guide bone regeneration.

The management and treatment of bone defects is a major clinical problems in the field of periodontology and oral implantology^1^,^2^. Currently, guided bone regeneration (GBR) is applied to restore alveolar bone, a process in which a GBR membrane is used to isolate periodontal bone defects from gingival connective tissue, so that newly formed bone can grow along the defect margins of alveolar bone^3^,^4^. However, a major issue with current GBR membranes is that they are susceptible to infection due to their exposure to pathogens colonization at wound sites in the process of bone healing, which decreases the amount of regenerated bone^5^,^6^. Clinically, antibiotic ointments are commonly applied to avoid the infection after GBR application, which increases the number of follow-up visits. Hence, it is necessary to fabricate a novel antibacterial GBR membrane for preventing infection and enhancing bone regeneration.

The antibacterial GBR membranes were prepared by loading antibacterial agents such as chlorhexidine, tetracycline, amoxicillin and minocycline^5^,^7^,^8^,^9^. Of these antibiotics, minocycline (7-dimethylamino-6-dimethyl-6-deoxytetracycline) is a semi-synthetic tetracycline derivative that has been used to inhibit both Gram-positive and Gram-negative bacteria by inhibiting bacterial protein synthesis through its binding to bacterial 30S ribosomal subunits^10^. Minocycline has also proven effective as a antimicrobial agent in periodontal therapy by inhibiting matrix metalloproteinases (MMPs), which can enhance bone formation, diminish bone resorption and decrease connective tissue breakdown^11^,^12^,^13^. An hydroxyapatite-gelatin-minocycline nanocomposite has been prepared and used to demonstrate that minocycline can be released from the composite particles slowly over 2 weeks *in vitro*^12^. Antibiotics, loaded into nanoparticles, can be released slowly and maintain necessary concentrations in the locality. Thus, nanoparticles loaded with minocycline, which is an appropriate candidate antibacterial drug incorporated into the GBR membrane to prevent infection, should have a significant effect on bone regeneration.

As described in our previous study, an asymmetric chitosan GBR membrane was prepared and showed the ability to help bone regeneration^14^. More recent studies have paid much attention to developing GBR membranes with asymmetric structures^15^,^16^,^17^, but few works have focused on developing an asymmetric GBR membrane loaded with drugs. Generally, the dense layer of an asymmetric membrane is designed to prevent fibrous connective tissue from invading defect spaces, and the loose layer that directly contacts the bone defect spaces is beneficial for osteoblasts adhesion and blood clots stabilization, which guides bone regeneration. In this study, we designed an asymmetric membrane, that has a dense layer acting as a barrier and a loose layer loaded with nanoparticles of minocycline as a carrier for drug release.

It has been shown that glycosaminoglycans and collagen types I and III are the major components of periodontal extracellular matrix^18^,^19^. Chitosan, a deacetylated derivative of chitin, is composed of *N*-acetyl glucosamine and glucosamine, and is structurally similar to the glycosaminoglycans in the extracellular matrix. Since chitosan contains a number of free amine groups^20^,^21^, it is easy to make chitosan into nanoparticles using ionic cross-linking. Thus, chitosan was chosen to prepare the loose layer of the asymmetric membrane and the minocycline-carrying nanoparticles.

Collagen, the material of the dense layer for the asymmetric membrane, is commonly found in the bone, skin, tendon, and connective tissue of animals^22^. Due to its excellent biocompatibility and biodegradability, collagen is a promising scaffolding material for tissue engineering^23^. Additionally, collagen membranes have been used in GBR surgery and have achieved satisfactory clinical results. However, it is difficult to load drugs into collagen membranes. Moreover, collagen is rapidly degraded when in contact with body fluids or cell-culture media. Cross-linking is an effective method to modify the biodegradation rate of collagen-based scaffolds^23^. Additionally, chitosan has a great number of amino groups in its molecular chain, which can act as a bridge via ionic cross-linking to enhance the physical properties of collagen-based membranes^24^. Thus, in this study, chitosan and collagen were chosen as the main materials to fabricate the membranes.

The objective of this study was to develop asymmetric collagen/chitosan membrane loaded with chitosan nanoparticles of minocycline. We hypothesized that a collagen/chitosan membrane can prevent infection through the local delivery of minocyline, while at the same time keeping soft tissue from invading bone defects, facilitating bone regeneration. This study investigated the morphology, biodegradation, drug release, antibacterial activity, cytocompatibility and bone regeneration ability of the membranes.

## Results

### Characterization of the CCM membrane

**Figure 1:**
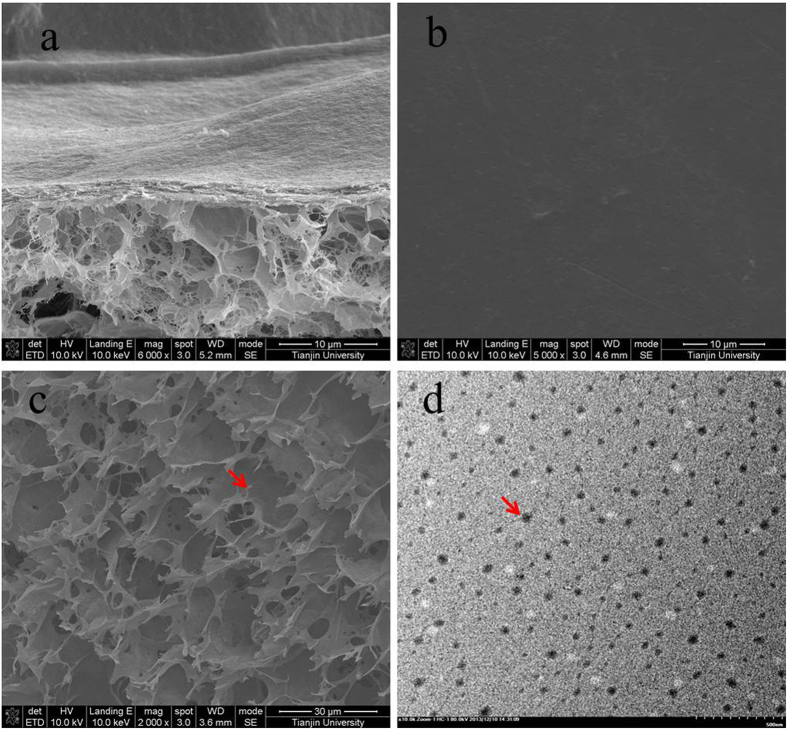
SEM images showing the morphologies of the cross-section (**a**), dense layer (**b**), and loose layer (**c**) of an asymmetric porous chitosan membrane. TEM image of chitosan nanoparticles of minocycline (**d**). The red arrow indicates the nanoparticles.

As shown in Fig. 1(a), the cross-section of the asymmetric collagen chitosan membranes loaded with minocycline (CCM membrane) presented an asymmetric structure, including a dense collagen layer and a loose chitosan layer. The surface morphology of the collagen layer and the chitosan layer are shown in Fig. 1(b,c). Moreover, nanoparticles appeared on the surface of the chitosan layer as expected. The TEM images also showed the morphological properties of the nanoparticles, which had smooth surfaces, nearly spherical shapes, and a size range of approximately 30–70 nm (Fig. 1(d)).

**Figure 2:**
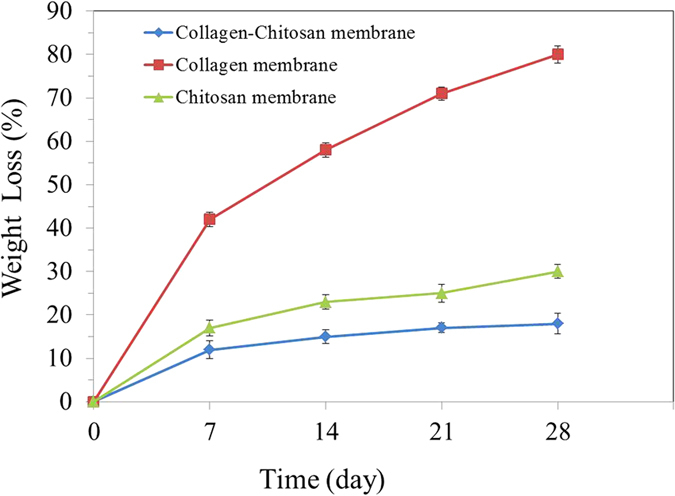
Weight loss of membranes during different soaking intervals (*n* = 3).

**Figure 3:**
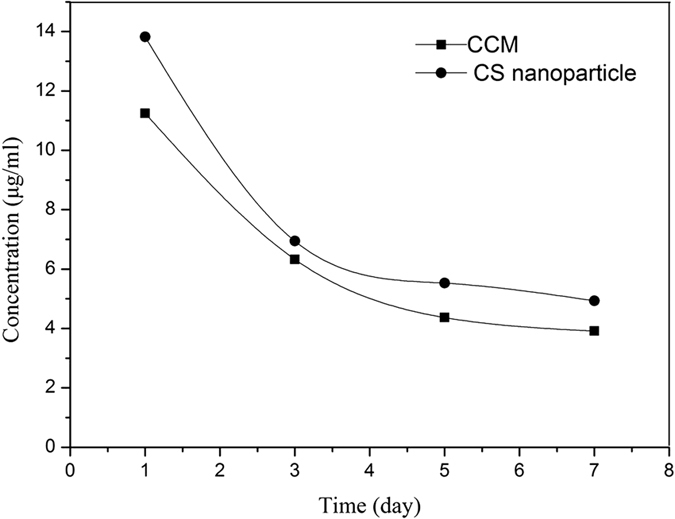
Release curve of minocycline from the CCM membrane and chitosan nanoparticle at 1, 3, 5, 7 days. (*n* = 3).

The results of the *in vitro* degradation rate of the membranes are shown in Fig. 2. The CCM membrane presented lower degradation rate than that of the chitosan membrane and the collagen membrane, which indicated that the cross-linked membranes maintained a low rate of degradation even after 28 days of soaking. All membranes showed weight losses that increased with increasing immersion time. The release of minocycline from the CCM membrane and chitosan nanoparticle is shown in Fig. 3. An initial burst was observed on the first day, and until the seventh day, both the membranes and microspheres have minocycline sustained release.

### Antibacterial activity of the CCM membrane

**Figure 4:**
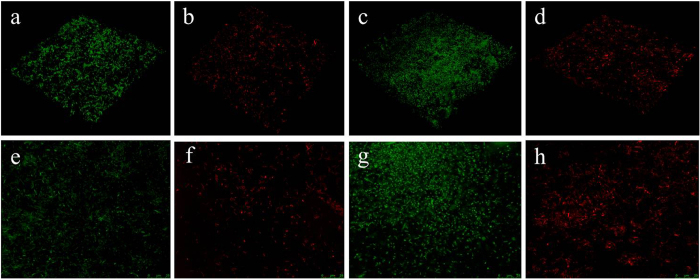
Characterization of the antibacterial activity of the CCM membrane and control membrane without minocycline against *Porphyromonas gingivalis* and *Fusobacterium nucleatum*. LSCM images are shown for the control membrane (**a,e**) and the CCM membrane (**b,f**) against *Fusobacterium nucleatum*, the control membrane (**c,g**) and the CCM membrane (**d,h**) against *Porphyromonas gingivalis* (green staining indicated live bacteria and red staining indicated dead bacteria).

The antibacterial efficiency of the CCM membrane was assessed against *Porphyromonas gingivalis* (ATCC No. 33277) and *Fusobacterium nucleatum* (ATCC No. 10953). The results showed that dead bacteria (red staining) were detected on the CCM membrane, whereas live bacteria (green staining) were observed on the membrane without minocycline (Fig. 4). The bacteriostatic rates of CCM membrane against *Porphyromonas gingivalis* and *Fusobacterium nucleatum* were 95.3% and 92.1%, respectively.

### Biocompatibility of the chitosan membrane

**Figure 5:**
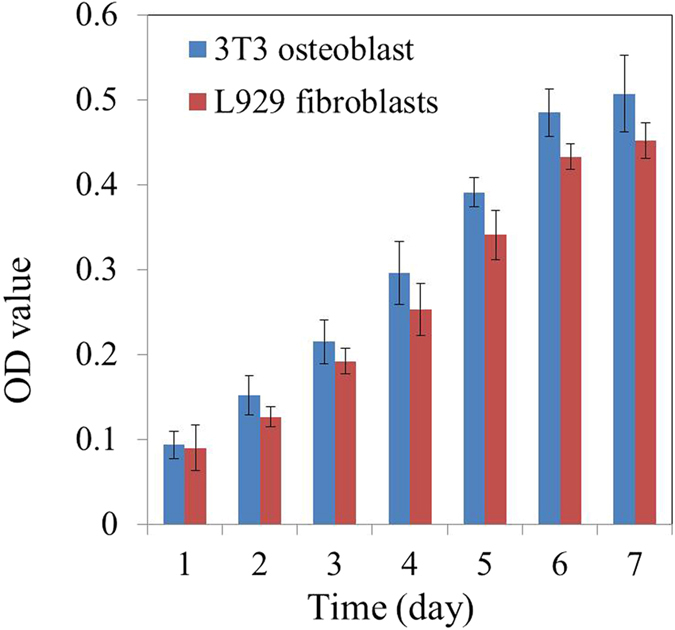
MTT assay results of osteoblasts and fibroblasts seeded on the surfaces of the chitosan layer and the collagen layer, respectively.(*n* = 3).

**Figure 6:**
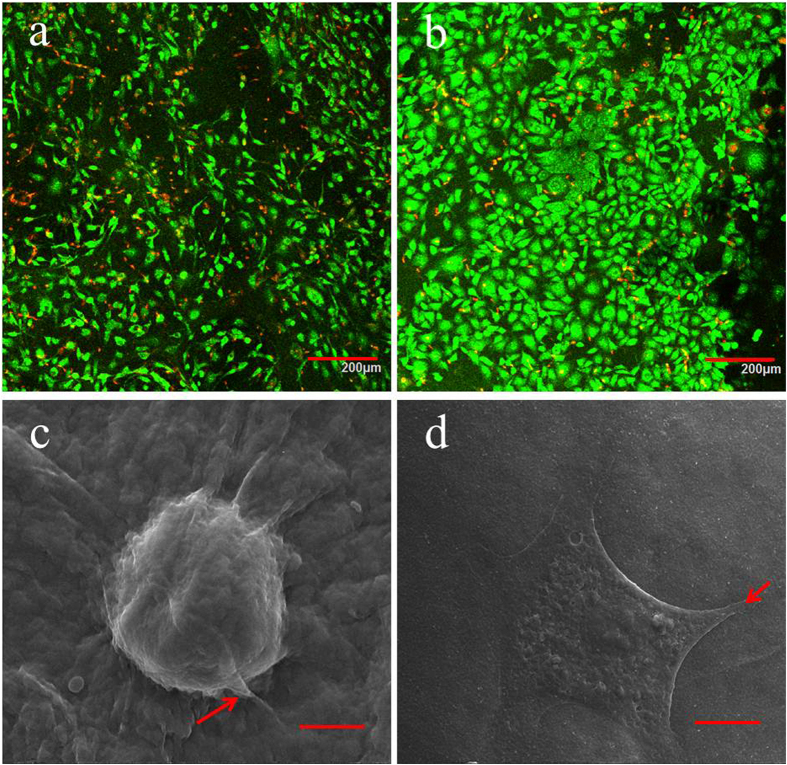
LSCM images of osteoblasts attached to the chitosan surface (**a**) and fibroblasts attached to the collagen surface (**b**) after culturing for 24 h (green staining indicated live cells and red staining indicated dead cells); SEM images of osteoblasts attached to the chitosan surface (**c**) and fibroblasts attached to the collagen surface (**d**) after culturing for 24 h. The red arrow indicates the discrete filopodia.

Figure 5 shows the MTT assay results of the osteoblasts and fibroblasts seeded on the chitosan surface and the collagen surface of the CCM membranes after 1, 4, 7 and 10 d. After 24 h, the cells rapidly proliferated and presented an increasing trend, suggesting that the CCM membranes possessed good biocompatibility. On the seventh day, the cells in both groups grew slowly, suggesting that the cells came into confluence, which resulted in contact inhabitation. The osteoblasts and fibroblasts on the surface of CCM membrane were stained by the Live/Dead Cell Double Staining Kit and characterized using LSCM (Fig. 6(a,b)). The representative images of LSCM showed that after culturing for 24 h, the osteoblasts and fibroblasts on the CCM membranes presented favorable cell morphology, indicating that the CCM membranes could maintain satisfactory cell viability.

SEM micrographs of the osteoblasts on the chitosan surface and the fibroblasts on the collagen surface are shown in Fig. 6(c,d). After 24 h of seeding, the osteoblasts showed a round shape, and were anchored to the chitosan surface by discrete filopodia, whereas the fibroblasts had fully spread, presenting a flat star-like shape. The cells adhered to the surface of the CCM membrane firmly, suggesting that the CCM membrane had satisfactory cytocompatibility.

### Bone regeneration behavior

**Figure 7:**
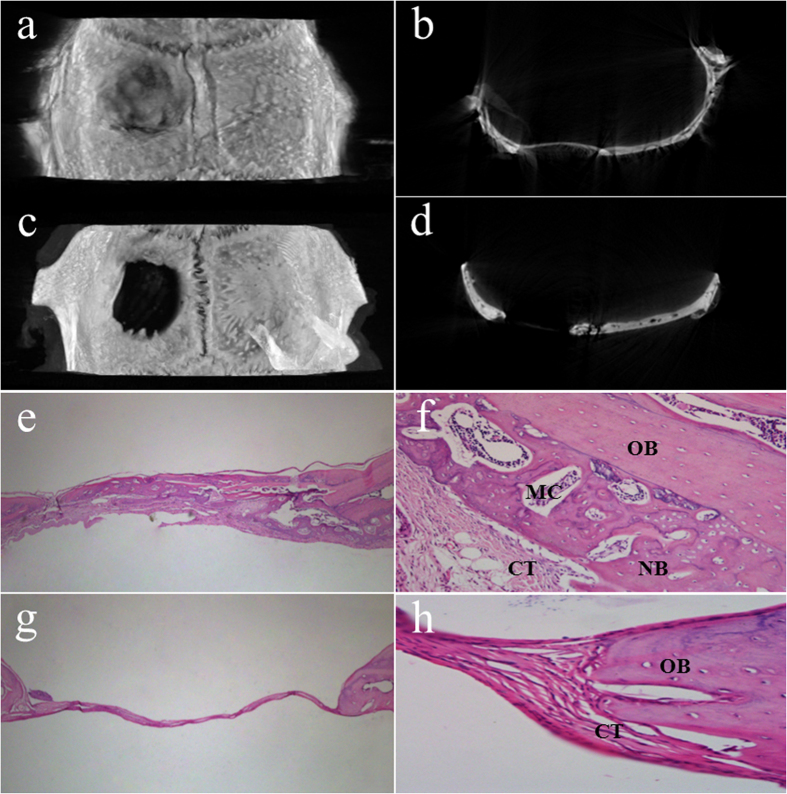
3D reconstruction and sagittal surface images of bone defects covered with CCM membrane (**a,b**) and without membrane (**c,d**); histological sections of bone defects and surrounding tissue, covered with CCM membrane (e × 40, f × 400) and without membrane (g × 40 and h × 400); OB, old bone; NB, new bone; CT, connective tissue; MC, marrow cavity.

The representative 3D reconstruction images of the repaired bone defects of rats obtained by Mirco-CT scanning are shown in Fig. 7(a,c). After 4 weeks of healing, substantial new bone formation was observed in both 3D images and on the sagittal surface for the CCM membrane group (Fig. 7(a,b)). However, in the control group, an obvious defect was left (Fig. 7(c,d)). The comparisons indicated that the CCM membrane could guide bone regeneration.

The images of histological sections of rat calvarial defects are shown in Fig. 7(e–h). In the control group (no membrane used), thin, loose connective tissue invaded the bone defects, which suppressed the formation of new bone. By contrast, in the CCM membrane groups new bone formation appeared beneath the membrane from both the periphery and the dural surfaces of the defect (Fig. 7(e)). No significant inflammatory reactions were revealed in the specimens, and the membranes were almost degraded. As shown in Fig. 7(f,h), the defect sites were observed at high magnification for the histomorphometric analysis. Newly formed bone containing bone marrow and osteocyte cells is shown in Fig. 7(f). However, in the control group, connective tissue grew into the bone defect area and obstructed bone formation (Fig. 7(h)). These results indicated that the CCM membrane could act as a physical barrier for the GBR technique.

**Figure 8:**
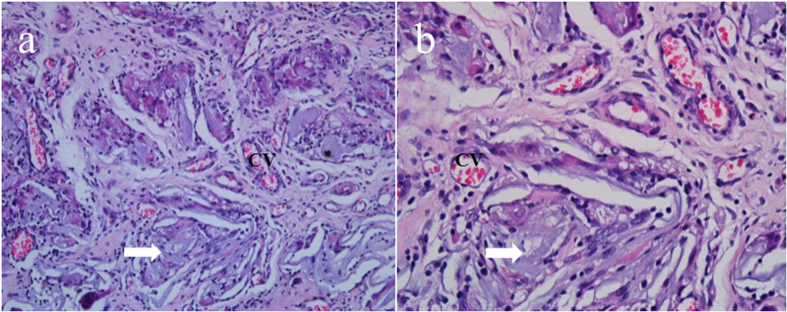
Histological sections of CCM membrane implanted after 4 weeks (a × 100 and b × 400); CV, capillary vessels; white arrow, degradable CCM membrane.

After implanting for 28 days, the integrated morphology and structure of the CCM membrane could not be observed. The membrane was almost completely degraded and integrated well with adjacent fibrous tissue (Fig. 8(a,b)). In addition, a large number of small blood vessels was observed around the degraded membrane, which was necessary for the construction of a large amount of tissue.

## Discussion

Many studies on the technical development of GBR membranes have focused on membrane biodegradation, biocompatibility, space maintaining ability, clinical manageability and bone regeneration ability^25^. However, the exposure of the membrane is a commonly observed phenomenon in GBR application and provides an environment for bacterial adherence and multiplication^26^. Currently, little efforts has been spent on improving the antibacterial function of barrier membranes. Minocycline loading electrospun chitosan membranes have been shown to reduce early bacterial contamination of GBR graft sites^5^. Furthermore, minocycline has also proven to be an effective antimicrobial agent in periodontal therapy by inhibiting MMPs, a process in which both direct and indirect mechanisms seem to be involved^27^. Direct inhibition appears to be mediated by tetracycline interactions with metal ions within the enzyme, whereas indirect mechanisms may be involve the inhibition of mRNA expression and synthesis of MMPs^28^,^29^,^30^. In addition, some research has reported that minocycline was active against methicillin (meticillin)-sensitiveand -resistant strains of Staphylococcus aureus^31^,^32^. However, in this study, we prepared a novel collagen/chitosan membrane loaded with minocycline, which showed antibacterial activity against P*orphyromonas gingivalis* (ATCC No. 33277) and *Fusobacterium nucleatum* (ATCC No. 10953), which are the main pathogenic bacteria of periodontitis.

A local antibiotic delivery system can release loaded drugs slowly and maintain an effective concentration in the vicinity of the wound for some time^33^. In this study, chitosan nanoparticles were selected as carriers to deliver minocycline, and ionic gelation using TPP was carried out. In this study, when minocycline was released from the chitosan nanoparticle loaded in the CCM membrane, the released concentration on the first day was on the higher side of the MIC range, and until the seventh day the concentration was maintained at over 3.9 μg/ml (Fig. 3). It should be noted that the actual amount of released drug at the wound site may be related to the rate of release and equilibrium concentration of the drug^34^. Another point of concern regarding drug-releasing GBR membranes is that the released drugs should not interfere with cellular activity but should show adequate activity against bacteria. The membrane prepared in this study showed satisfactory cytocompatibility (Figs 5 and 6) and antibacterial activity (Fig. 4).

The critical-size rat cranial defect model was chosen to investigate the bone regeneration behavior of the CCM membranes. The critical size defects were originally defined by Schmitz and Hollinger in 1986 as “the smallest size intraosseous wound in a particular bone and species of animal that will not heal spontaneously during the lifetime of the animal.”^35^ In this study, critical size defects of 8 mm in diameter were selected in animal experiments with 4 weeks healing period. The newly created bone was observed in the CCM membrane groups in both Micro-CT images and HE staining images (Fig. 7(a,c)), suggesting that the loose chitosan layer of the membranes is osteoconductive and that the dense collagen layer can act as a physical barrier. Furthermore, in the high magnification image of the HE staining, the marrow cavity structures were observed (Fig. 7(f)), which may contain mesenchymal stem cells and capillary vessels, showing the potential of osteoinduction^36^. A vascularized environment is necessary for the construction of bone tissue. In this study, a large number of small blood vessels were found in the degraded membranes (Fig. 8(b)), which can transport nutrient and stem cells during the healing period.

After implanting for 28 days, degradation of the CCM membrane was observed (Fig. 8(a)). Many of the earlier studies used nondegradable expanded polytetrafluorethylene (ePTFE) membranes to guide bone regeneration successfully^37^. However, a major advantage of the degradable membranes made of materials such as collagen and chitosan is that a second surgical intervention can be avoided. In clinical practice, it is necessary for the GBR membrane to maintain a balance between the degradation rate of the membrane and the rate of tissue regeneration^38^. The animal experiment in this study showed the degradation of the CCM membrane (Fig. 8) and the regeneration of bone tissue (Fig. 7). However, the degradation of the CCM membrane *in vitro* did not match that *in vivo*. One possible reason for this finding is that SBF was applied *in vitro*, which has some differences compared with the composition of body fluid *in vivo*.

The CCM membranes prepared in this study showed satisfactory bone formation ability, biodegradability, and antibacterial ability. Although the practical use of the CCM membrane will be determined by further clinical studies, these results suggest that these membranes have the potential to be used as a novel GBR membrane for clinical application. A future study will investigate the effects of stem cells or growth factors loaded into the CCM membrane on tissue regeneration.

## Conclusion

The asymmetric antibacterial collagen/chitosan GBR membrane can be fabricated by loading minocycline encapsulated chitosan nanoparticles, phase separation and lyophilization. The resulting CCM membrane has an asymmetric structure that include a loose chitosan layer and a dense collagen layer; the former can promote osteoblasts adhesion and inhibit bacteria colonization, and the latter can promote fibroblasts adhesion and prevent fibrous tissue infiltrating into the bone defects, thereby guiding bone regeneration. The asymmetric structure, biodegradability, biocompatibility, antibacterial activity and guided bone regeneration ability of the CCM membrane may fulfill clinical requirements.

## Materials and Methods

### Materials

Chitosan (87% deacetylated), ice acetic acid, sodium tripolyphosphate (TPP) and ethylenediaminetetraacetic acid (EDTA) were purchased from Life Science Products & Services (Shanghai, China). Brian Heart Infusion (BHI) medium, agar, minocycline, EDC (1-Ethyl-3-(3-dimethylaminopropyl)-carbodiimide), NHS (N-hydroxysuccinimide), MTT (3-[4,5-dimethyl-thiazol-2-yl]-2,5-diphenyl-tetrazoliumbromide) and a Live/Dead Cell Double Staining Kit were all purchased from Sigma (St. Louis, MO, USA). Dulbecoco’s Modified Eagle Medium (DMEM), horse serum, penicillin, and streptomycin were all purchased from HyClone (Logan,Utah, USA). Hematoxylin-eosin (H&E) was purchased from Baso (Zhuhai, China). *Porphyromonas gingivalis* (ATCC No. 33277) and *Fusobacterium nucleatum* (ATCC No. 10953) were obtained from the China General Microbiological Culture Collection Centre. Distilled water (ultrapure grade, <18 mΩ) was produced by the Mili-Q puri-fication system (EMD Millipore Corporation, Billerica, MA, USA).

Collagen was isolated from mouse-tail tendons using the acetic acid dissolution method. Briefly, mouse tails were washed and disinfected (in 2% sodium hypochlorite) before the dissociation of the tail tendons. After removing the surrounding fat and muscle, the tendons were cut into small thin pieces and dissolved in 0.5 M acetic acid at 4 °C for 48 h. Subsequently, the suspension was centrifuged, and redissolved in 0.1 M acetic acid. After the pH of the solution adjusted to 5.0 with 0.1 M NaOH, EDC/NHS was used as a crosslinker. Subsequently, 22.4 mg EDC and 5.6 mg NHS were added to 10 mL collagen solution in batches at room temperature to react for 10 minute, and the collagen solution was then obtained.

### Preparation of the nanoparticles loaded with minocycline

Chitosan nanoparticles were prepared based on ionic gelation in which polyanionicsodium TPP can interact with the cationic chitosan by electrostatic forces^20^. Briefly, chitosan was dissolved in 1% (v/v) acetic acid to obtain a 0.2% (w/v) chitosan solution, and minocycline with a concentration of 15 μg/ml was added to the chitosan solution (10 ml) with stirring. Subsequently, TPP (0.2% (w/v) distilled in water) was added dropwise into the chitosan minocycline mixture under vigorous magnetic stirring. The resulting mixture was stirred for another 20 min and then centrifuged at 15,000 r/min for 15 min. Subsequently, the precipitate was suspended in distilled water and centrifuged again. Usually, 20 μg of minocycline-loaded chitosan nanoparticles were obtained by freeze-drying.

### Fabrication of asymmetric collagen chitosan membranes

Chitosan nanoparticles of minocycline 20 μg were added into 10 mL chitosan solution (2% (w/v)) to prepare the chitosan mixture. Afterward, 2.5 mL of the chitosan mixture was slowly poured on a polytetrafluoroethylene mold (15 mm in diameter, 1.5 mm in depth) at room temperature to obtain an even liquid film. Then, the liquid film was pre-heated at 45 °C for 1 h for dry phase separation before immersion into the sodium hydroxide solution for wet phase separation. Subsequently, the obtained chitosan membrane was washed repeatedly with the distilled water until the pH value was neutral. Afterwards, the crosslinked collagen solution was poured on the obtained chitosan membrane, pre-heated for 2 h to form a collagen/chitosan membrane, and then frozen and lyophilized to generate asymmetric collagen chitosan membranes loaded with minocycline (CCM). Pure chitosan membrane or pure collagen membrane was prepared by the method mentioned above. Pure chitosan membrane was prepared by phase separation method with 2% (w/v) chitosan solution, meanwhile, pure collagen membrane was prepared by lyophilization with non-crosslinked collagen solution.

### Characterization of collagen chitosan membranes

#### Morphological observation

A scanning electron microscope (SEM; Nova NanoSEM 430, FEI, Netherland) was used to observe the surface and cross-section morphology of the CCM membranes. The cross-section of the membrane was prepared by fracturing the sample with liquid nitrogen before morphology observations. Then, the samples were sputter-coated with gold in argon atmosphere using a sputter coater (K575XD, Emitch, England).

The morphological characteristics of chitosan nanoparticles of minocycline were examined by a transmission electron microscope (TEM, JEM-2100F, Japan). The samples were immobilized on copper grids (400 mesh size). They were dried at room temperature, and then examined using TEM without being stained.

#### *In vitro* biodegradation

Degradation behavior was evaluated by incubating the specimens (15 mm in diameter) in 20 mL of simulated body fluid (SBF) at 37 °C. After soaking for 7, 14, 21, and 28 days, the specimens were taken out of the degradation medium, washed with distilled water and dried. The degradation rate is expressed as a percentage, calculating the relative weight loss of the specimens.

#### *In vitro* release of minocycline

The CCM membrane (15 mm in diameter) and 5 μg of minocycline-loaded chitosan nanoparticles were incubated in 1 mL of PBS (pH 7.4) in a constant temperature oscillator (100 rpm) at 37 °C to determine the release of minocycline. At each time intervals (1, 3, 5, and 7 days), the supernatant was collected, and ultraviolet spectrophotometry (BioSpectrometer, Eppendorf, Germany) was used to measure the amount of minocycline released at 348 nm (*n* = 3). Then, all the PBS was collected and replaced with a fresh one. The concentrations of minocycline was calculated through comparisons with a standard curve.

#### *In vitro* antibacterial activity

The antibacterial activity of the CCM membrane was examined against *Porphyromonas gingivalis* and *Fusobacterium nucleatum*. The membranes were sterilized using gamma rays (cobalt-60) with a dose of 20 kGy for 6 h (Chinese Academy of Medical Sciences Institute of Radiation Medicine). *Porphyromonas gingivalis* and *Fusobacterium nucleatum* was cultured in an anaerobic chamber (N_2_: 80%, H_2_: 10%, CO_2_: 10%) at 37 °C and in freshly prepared BHI agar plates and supplemented with 1% yeast extract for 16 h at 37 °C. Then, the bacteria were collected and resuspended at 1 × 10^7^ cfu/ml as a primary inoculum.

Live/dead bacterial double staining was used to distinguish dead bacteria (red staining) from live bacteria (green staining) using laser confocal microscopy (LSCM) (Fv-1000, Olympus, Japan). First, 2 mL of primary inoculum were inoculated on the membranes in a 12-well plate. After a 24 h culturing period, the membranes were taken out of the plates, washed with PBS three times, placed on new plates, and then stained using the Live/dead staining kit. Subsequently, several decimal solutions were sampled from the 12-well plate after the exposure of bacteria to the CCM membranes. Then, 30 μL of the diluted bacterial solution was spread on the BHI agar plates and incubated at 37 °C. After 24 h, the numbers of the surviving colonies were counted. Then bacteriostatic rate can be obtained from the following equation:


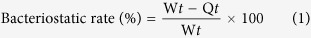


where W*t* is the average number of colonies counted for the control that had not been exposed to the CCM membranes; and Q*t* is average the number of colonies obtained from each tested sample.

#### Evaluation of biocompatibility *in vitro*

MC3T3-E1 osteoblasts and L929 fibroblasts were used to evaluate the biocompatibility of the CCM membranes. The sterilized CCM membranes were cut into discs with 15 mm in diameter matching the well size of a 24-well plate and pre-wetted with complete growth medium (DMEM with 10% fetal bovine serum, 100 mg/mL of streptomycin, and 100 U/mL of penicillin) before adding the cells. Subsequently, 10,000 osteoblasts or fibroblasts in 1000 μL of medium were seeded onto the chitosan surface or collagen surface of the sterilized membranes, and placed in a new 24-well plate, respectively. The membranes were maintained in a 5% CO2 incubator at 37 °C. At various culturing intervals, 10 μL/well of MTT (5 mg/mL) and 90 μL/well of fresh medium were added to the 24-well plates to replace the original culture medium. These plates were then incubated in the 5% CO2 incubator at 37 °C for 4 h. Subsequently, the supernatant was discarded, and 110 μL/well of dimethyl sulfoxide (DMSO) were added to the plates for 30 min. Then, an aliquot of the resulting solution (80 μL) was transferred to a 96-well plate, and the absorbance was measured at 490 nm using a Microplate Reader (RT-6000, Rayto, USA).

For morphological observation, the CCM membranes were taken out from the plates after a 24 h culturing period. Then the cells on the membranes were washed with PBS three times. Half of the membranes were stained using the Live/Dead Cell Double Staining Kit and observed using laser confocal microscopy (LSCM) (Fv-1000, Olympus, Japan). The others were fixed using 2.5 vol% glutaraldehyde in 0.1 M sodium cacodylate buffer (pH 7.4) for 24 h at 4 °C. Subsequently, the cells were washed with PBS three times, dehydrated using graded ethanol (30, 50, 75, 95, and 100%) and then dried before sputtered with gold. The morphology of the adhered cells were observed using SEM.

#### Animals and operation procedure

To investigate the bone regeneration behavior of the CCM membranes, the skull defect model of Sprague-Dawley rats was established. The institutional ethics committee of the Tianjin Medical University approved the use of Sprague-Dawley rats for the experiments conducted in this study, and all experiments were performed in accordance with relevant guidelines and regulations. The rats (320~360 g, male, 8 weeks) were divided into control and CCM membrane groups. The rats were anesthetized by intraperitoneal injection of chloral hydrate (1 mL/330 g Yulong, China). After exposure of the parietal calvarium, a full-thickness skull defect (8 mm in diameter) was generated with a trephine bur driven by a handpiece. Subsequently, the hydrated CCM membranes (15 mm in diameter) were placed on the defects. In the control group, the defects were covered without the membrane. The periosteum and skin were sutured using 5–0 suture line.

To evaluate the degradation and inflammation of the CCM membrane *in vivo*, a 20 mm area of the rat dorsal skin was cleaned and shaved. The CCM membranes (5 mm in diameter) were inserted through a 5 mm incision on the dorsal lower right quadrant and secured using one nonabsorbable nylon suture stitch.

#### Microcomputed tomography (Mirco-CT) scanning evaluation

At the end of 28 days, the animals underwent euthanasia by injecting an overdose of sodium pentobarbital. Then, the bone defects with surrounding cranial tissues and the membranes with soft tissue were removed from the bodies. The specimens were fixed in 10% neutral-buffered formalin. A desktop X-ray Micro-CT scanner (SkyScan 1174v2, SkyScan, Kontich, Belgium) was used to determine the bone mineral density of the bone defects. The samples were scanned with a spatial resolution of 6.55 μm and their projection images were collected at 50 kV and 800 A using 360° rotation with 0.7° per projection step. The files were reconstructed using a microtomographic analysis software, and three-dimensional (3D) images were acquired.

#### Histological preparation and evaluation

The tissue specimens were decalcified in 17% EDTA solution (except the specimens of membranes with soft tissue), dehydrated in an ascending graded series of alcohol, and embedded in paraffin. A series of 5 μm transverse sections in the center of the bone defects or the membrane with soft tissue were prepared and stained with H&E for observation by light microscopy (BX51, Olympus, Japan).

## Additional Information

**How to cite this article**: Ma, S. *et al*. Asymmetric Collagen/chitosan Membrane Containing Minocycline-loaded Chitosan Nanoparticles for Guided Bone Regeneration. *Sci. Rep*. **6**, 31822; doi: 10.1038/srep31822 (2016).
